# Fertility Sparing Treatments in Endometrial Cancer Patients: The Potential Role of the New Molecular Classification

**DOI:** 10.3390/ijms222212248

**Published:** 2021-11-12

**Authors:** Anna Franca Cavaliere, Federica Perelli, Simona Zaami, Marco D’Indinosante, Irene Turrini, Marco Giusti, Giuseppe Gullo, Giuseppe Vizzielli, Alberto Mattei, Giovanni Scambia, Annalisa Vidiri, Fabrizio Signore

**Affiliations:** 1 Azienda USL Toscana Centro, Gynecology and Obstetric Department, Santo Stefano Hospital, 59100 Prato, Italy; annafranca.cavaliere@uslcentro.toscana.it (A.F.C.); marco.dindinosante@uslcentro.toscana.it (M.D.); irene.turrini@uslcentro.toscana.it (I.T.); 2 Azienda USL Toscana Centro, Gynecology and Obstetric Department, Santa Maria Annunziata Hospital, 50012 Florence, Italy; marco1.giusti@uslcentro.toscana.it (M.G.); alberto.mattei@uslcentro.toscana.it (A.M.); 3 Department of Anatomical, Histological, Forensic and Orthopedic Sciences, Sapienza University of Rome, Viale Regina Elena 336, 00161 Rome, Italy; simona.zaami@uniroma1.it; 4 Azienda Ospedaliera Ospedali Riuniti (AOOR) Villa Sofia Cervello, IVF Public Center, University of Palermo, 90100 Palermo, Italy; gullogiuseppe@libero.it; 5 Clinic of Obstetrics and Gynecology, Dipartimento di Area Medica (DAME), University Hospital of Udine, University of Udine, 33100 Udine, Italy; giuseppe.vizzielli@asufc.sanita.fvg.it; 6 Gynecologic Oncology Unit, Fondazione Policlinico Universitario A. Gemelli, Istituto di Ricovero e Cura a Carattere Scientifico (IRCCS), 00168 Rome, Italy; giovanni.scambia@policlinicogemelli.it (G.S.); annalisavidiri@gmail.com (A.V.); 7 Obstetrics and Gynecology Department, Unità Sanitaria Locale (USL) Roma 2, Sant’Eugenio Hospital, 00144 Rome, Italy; fabrizio.signore@aslroma2.it

**Keywords:** endometrial cancer, molecular biology, fertility sparing, obstetric outcomes, pregnancy

## Abstract

Endometrial cancer is the most frequent gynecological malignancy, and, although epidemiologically it mainly affects advanced age women, it can also affect young patients who want children and who have not yet completed their procreative project. Fertility sparing treatments are the subject of many studies and research in continuous evolution, and represent a light of hope for young cancer patients who find themselves having to face an oncological path before fulfilling their desire for motherhood. The advances in molecular biology and the more precise clinical and prognostic classification of endometrial cancer based on the 2013 The Cancer Genome Atlas classification allow for the selection of patients who can be submitted to fertility sparing treatments with increasing oncological safety. It would also be possible to predict the response to hormonal treatment by investigating the state of the genes of the mismatch repair.

## 1. Introduction

Endometrial cancer (EC) occurs after 50 years of age in approximately 80% of cases, affecting approximately 20% of women in premenopausal age, and only 5% of them under 40 years old [1,2].

The actual delay in childbearing age is one of the reasons why EC is now diagnosed before a first pregnancy more frequently, with respect to the past.

In endometrioid EC, standard management involves total hysterectomy and bilateral salpingo-oophorectomy, leading to very high cure rates of 93% in low-risk disease [3].

Medical treatment and uterine sparing management are now accepted as reasonable short-term alternatives to definitive surgical management in highly selected patients [4]. The fertility sparing treatment (FST) in EC includes hysteroscopic resection and/or curettage, in combination with hormonal therapy with progestin. In this setting, complete remission rates of 50–75% have been reported [5,6,7], and strict follow-up with hysteroscopic evaluation and endometrial sampling is recommended. The obstetric outcome of EC patients is affected by several factors, many of which are the same risk factors associated with the development of EC itself, such as polycystic ovary syndrome (PCOS), infertility, and obesity.

The new molecular classification based on genetic risk factors reported by the European Society of Gynaecological Oncology (ESGO), the European Society for Radiotherapy and Oncology (ESTRO), and the European Society of Pathology (ESP) consensus published in 2021 seems to impact on both the prognosis and the FST response of these patients [8,9]. The present review aims to establish the basis for further retrospective and prospective studies to verify how the new molecular classification of EC could really improve the selection of young patients eligible for a fertility sparing program, to guarantee both their oncological safety and the success of FST response and subsequent pregnancy.

## 2. Fertility Sparing Treatments

### 2.1. Patients Selection

In the context of FST, the most important question asked is about which women can benefit from this type of treatment. Fundamental in the choice of this clinical path is to consider the patient’s desire to maintain fertility. The clinicians must also evaluate any significant medical comorbidity that may contraindicate a surgical treatment (obesity or chronic diseases), histological and molecular characteristics of the tumor, and, finally, the presence of family history or genetic syndromes. Usually, women of reproductive age diagnosed with EC have a low-grade, early-stage tumor [1,10,11], and they represent the ideal group for FST. The risks associated with this method are represented by a missed diagnosis of metastases to the ovaries or lymph nodes, and this absence could be covered by a careful surgical exploration, including a surgical study of possible metastatic lymph nodes. Obviously, a rigorous follow-up is essential for such patients. It is important to evaluate each patient’s demographic and clinical characteristics, including age, comorbidities, and obesity. Elderly patients often present conditions that could contraindicate surgery and, therefore, these women would benefit from a conservative treatment allowing them to avoid the major surgical complications that are associated with surgery. In fact, these patients present an increased risk of intra- and post-operative complications that are associated with an increased operative stay [12,13,14]. In this group of frail patients, minimally invasive surgery is correlated with a lower intraoperative blood loss and reduced postoperative pain. In addition, recovery time is faster, therefore the hospital stay is shorter [15,16]. A meta-analysis of the LACE [17] and LAP2 [18] studies showed comparable survival outcomes between laparotomy and minimally invasive surgery in EC patients [19]. Obese women represent a high-risk subset of EC patients, with higher rates of peri-operative complications and longer hospital stays [20]. The rate of conversion from laparoscopic to laparotomy also increases as BMI increases, with 57% of patients with a BMI greater equal to 40 kg/m^2^ requiring surgical conversion [18].

Therefore, the use of alternative treatments that are safe and less invasive is critical for these EC patients, and certainly the results of prospective studies (such as the feMMe trial for obese patients [21]) will investigate this situation. Moreover, in the evaluation of the feasibility of FST, it is important to evaluate the intrinsic characteristics of the tumor, since only patients with endometrioid histotype, grade 1 and without myometrial invasion can benefit from this treatment [8].

A work by Leitao et al. [22] compared grade 1 tumors diagnosed preoperatively with dilatation and curettage (D&C) to those diagnosed via pipelle biopsy, and the results showed that fewer tumors diagnosed by D&C were upstaged at surgery than those diagnosed by pipelle biopsy (8.7% vs. 17.4%; *p* = 0.007). Based on the disparities of pathologists in differentiating between complex atypical endometrial hyperplasia and grade 1 EC [23,24], the precision of the histological diagnosis for FST may be enhanced by a pathological review. For evaluation of myometrial invasion, the combined use of MRI and transvaginal ultrasound appears highly effective. Finally, patients with EC occurring at a young age should be genetically studied for the possibility of a genetic predisposition. The risk due to familiarity for EC has already been evidenced by meta-analyses and population-based studies [25,26], and may include a multitude of conditions, including lifestyle and environmental choices that may be affected by genetic predisposition. In women diagnosed with Lynch syndrome, the risk of developing EC during their lifetime is estimated to be 40% to 60%, and is associated with germline mutations in the mismatch repair (MMR) genes, MLH1, MSH2, MSH6, or PMS2 [27]. In a study of families with Lynch syndrome, the cumulative risk of EC by age 70 years was significantly higher among carriers of MLH1 mutations (54%), but this risk did not exceed 2% in women younger than 40 years of age [28]. As described in the National Comprehensive Cancer Network (NCCN-USA) guidelines for hereditary cancers, all women with EC should be genetically tested for MMR mutations, especially if diagnosed under 50 years of age, and if they are members of Lynch families [4].

### 2.2. Conservative Procedures

Conservative treatment of EC patients can be grouped in two paths: hormonal therapy, and hysteroscopic treatments. Hormonal therapy involves the use of oral progestins, such as medroxyprogesterone acetate (MPA) or megestrol acetate (MA), and, more recently, also levonorgestrel-releasing intrauterine devices (LNG-IUDs). Current evidence establishes administration of MPA at a dose of 400–600 mg/day, or MA at a dose of 160–320 mg/day for at least six months, and recommends performing a follow-up assessment of treatment response using D&C and imaging [8,29]. In contrast, the LNG- IUD consistently releases 52 mg of progestin for five years, following which there is a decrease in its release. In women undergoing FST, progestins showed satisfactory response rates (76–81%) in atypical hyperplasia and EC [6,30,31], and MA has shown higher remission rate compared to other hormonal treatments [30]. In a study by Park et al., in women aged ≤ 40 years with grade 1 endometrioid EC treated with daily oral MPA or MA, overlapping complete response rates (77.7%) were achieved after a median follow-up of 66 months; however, MPA correlated with a lower risk of recurrence. BMI ≥ 25 kg/m^2^ represents a failure factor that contrasts a complete response [5]. Although several papers do not report significant toxicity among patients treated with high-dose oral progestins, adverse events such as thrombophlebitis, pulmonary emboli, weight gain, hypertension, and headaches, may lead to low patient compliance [23,32,33]. For this situation, a solution is represented by the LNG-IUD, since it avoids the non-compliance associated with oral progestins, as well as the possible side-effects associated with their high-dose. A recent meta-analysis established that regression rates for the LNG-IUD and oral cyclic MPA treatment were comparable [34]. The Korean Gynecologic Oncologic Group disregarded the results of a prospective multicenter trial regarding the efficacy of LNG-IUD associated with oral MPA at 500 mg/day in patients with early-stage EC and age < 40 years [35]. Hysteroscopy also allows for more detailed and precise removal of disease from the surrounding margins and myometrium, providing a more accurate assessment of molecular and clinical features of the tumor through more adequate sampling of the disease than curettage. A trial by Alonso et al. [36] reviewed studies of EC patients with age < 40 years treated with initial hysteroscopic resection followed by hormone therapy for fertility sparing, and found that the complete response rate for patients with stage 1A grade 1 EC was 88.9%. A recent paper by Masciullo et al. evaluated the prognostic outcome of young women diagnosed with endometrial atypical hyperplasia (EAH)/endometrioid intraepithelial neoplasia (EIN) and early-stage EC (EEC) who were selected for fertility preservation, and treated with oral MA alone or combined with hysteroscopic resection. They found that hysteroscopic resection of EAH/EIN or EEC in combination with oral progestin therapy was significantly associated with shorter treatment duration to achieve complete response, and a longer time to relapse than treatment with progestin therapy alone [37]. In the FST, a primary role is played by the patient’s adherence to follow-up, and it is critically significant that the need for close follow-up and serial endometrial biopsies to assess response to treatment are understood and accepted by the patient. If so, current indications recommend evaluation at 3–6 months by endometrial biopsy [29,38]. It has been defined how long treatment should last to achieve a complete response; however, there is little evidence to indicate the presence of benefit beyond six months of therapy. A meta-analysis showed that the probability of remission after 6 months of treatment was lower than after 12 months of treatment (72% vs. 78%) [30]. Complete response (no evidence of progressive disease) is a good index for pregnancy planning purposes, and close follow-up should be kept in mind to diagnose pregnancy or relapse. Patients with partial response at 6 months of therapy may be advised to continue progestin treatment for approximately 3–6 months. In contrast, those who do not respond at 6-month follow-up and with persistence of disease should be referred for surgery, and then advised to undergo hysterectomy.

## 3. Molecular Classification and Its Impact on Fertility Outcomes

In 2013, a diagnostic classification supporting the tumor’s molecular biology was provided by The Cancer Genome Atlas (TCGA) Research Network with the paper titled “Integrated genomic characterization of endometrial carcinoma” [39]. The authors reported four prognostic categories, as follows: POLE ultra-mutated, microsatellite instability hypermutated, low copy-number tumor, and high copy-number tumor. Each group is characterized by specific mutations and different prognosis. The POLE ultra-mutated presents the most favorable prognosis, and is associated with a longer progression-free survival. Usually, this group is correlated with endometrioid histotype, and is linked with the subsequent mutated genes: POLE, PTEN, PIK3R1, PIK3CA, FBXW7, KRAS, and TP53 [40]. The hypermutated with microsatellite instability (MSI)/mismatch repair deficient (MMRd) group is characterized by an intermediate prognosis, and is also associated with endometrioid EC. In this group, the specific mutated genes are PTEN, KRAS, and ARID1A [40]. The MSI group denotes that the altered mechanisms of DNA MMR and four MMR genes (MLH1, MSH2, MSH6, or PMS2) are individuated whose inactivation leads to mutation accumulations called MSI through several mechanisms: insertions, deletions, point mutations, loss of heterozygosity, copy number changes, structural rearrangements, and methylation of a promoter gene [41]. The third group is the low copy number, which is characterized by an intermediate prognosis and linked to endometrioid EC. In this group mutated genes are CTNNB1 and PTEN [40], and the genome variation is due to duplication or deletion that changes the number of DNA base pairs. Lastly, the high copy-number is linked with an unfavorable prognosis and to serous histotype. This group is associated with genomic instability of the tumor and fast growth progression and invasion. The mutated genes are TP53, FBXW7, and PPP2R1A [40]. In this scenario, there is a growing interest towards the identification of risk factors and stratification of women based on the biology of the disease. Molecular markers can be combined with immunohistochemical examinations on tumor biopsy samples before planned treatment [42]. A review by Travaglino et al. evaluated the immunohistochemical markers that predict response to treatment, and showed markers from different pathways potentially involved in a good response or resistance to progestin therapy [43]. In this study, an indicator of good response was Dusp6, a marker of the mitogen-activated protein kinase (MAPK) signaling pathway, and its absent expression, the deficiency of MMR, as well as the involvement of PTEN, an activator of the mTOR/AKT/PI3K pathway, were important signs of potential therapy failure. This underlines a focus towards the pathways of MAPK and mTOR/AKT/PI3K, and not only towards progesterone and estrogen signaling. Another trial including women with EC has shown a higher incidence of early onset EC in patients with CTNNB1 somatic gene mutation, and, also, the accumulation of beta-catenin was inversely correlated with patients’ age [44]. Moreover, the overexpression of beta-catenin could be an index of treatment failure in FST [45]. In this setting, the CTNNB1 mutation is obtaining a major role as a molecular classifier of EC, especially in young patients [46]. Myers et al. revealed that patients with CTNNB1 mutation have a risk of recurrence which is nine times higher than in those without mutation [47]. Moreover, Kurnit et al. found that in stage I or II, the existence of either Tp53 mutations or CTNNB1 mutations was a risk factor for disease recurrence in presumed low-risk patients [48]. While Tp53 has relatively low copy number alterations, additional genes for risk stratification were PTEN, PIK3CA, ARID1A, and KRAS [39]. In fact, PTEN and PI3K/Akt/mTOR signaling pathways were associated with poor prognosis, but the association was only significant when models for risk stratification incorporated PTEN status and one of the molecular marker statuses outcomes [43,44,45,46,47,48,49]. In silico examination described new gene signatures associated with a worse overall survival in EC patients: CDK1, KIF2C, UBE2C, and TPX2.

Improving risk stratification for FST is one of the future targets, since the EC molecular classification and different molecular markers emerging are changing the risk profile assessment for patients, and not all stage IA, grade 1 patients present the same biology and associated outcomes. Indeed, Bosse et al. showed that the biological risk of grade 3 EC differs greatly if molecular classification is applied to them, as the grade 3 tumor group was made up of 12.9% of POLE ultramutated tumors, 20.7% of p53 abnormal tumors, 30.2% non-specific molecular profile (NSMP) tumors, and 36.2% of MMRd tumors, and POLE ultramutated and MMR-deficient remained independent prognostic factors for improved recurrence free survival in the grade 3 endometrioid cancer group [50]. The integrated clinical, pathological, and molecular tumor features were reported in TCGA and Proactive Molecular Risk Classifier for Endometrial Cancer (ProMisE) [39], and were summarized in a consensus published in 2021 [8]. Although the molecular classifiers are hopeful for the management of women with EC, available data now do not allow clinical applicability in order to extend the potential candidates for FST.

## 4. Obstetric Outcomes

The actual delay in childbearing age is one of the new emerging problems for the onco-gynecologist, since EC is diagnosed before the first pregnancy more frequently with respect to the past: 6.5% of women with EC are younger than 45 years old [51], and almost 70% of them are nulliparous at diagnosis [52]. Some young EC patients request a FST after adequate selection and well-informed process. In some surveys, “fertility” has been reported by patients as one of the most important determinants of their quality of life after treatment [53]. Less than half of the women affected by a gynecological cancer seem to receive adequate counseling regarding fertility preservation options. A multidisciplinary collaboration between gynecologic oncologists and fertility specialists might allow this gap to be reduced [54,55].

The pregnancy rate in women who underwent FST oscillated between 35–60% with hormonal treatment alone, but after a combined treatment based on hysteroscopic and hormonal therapy this pregnancy rate increased to approximately 70%. Moreover, hysteroscopic resection followed by progestogens is associated with a higher complete response rate, live birth rate, and lower recurrence rate than oral progestogens alone [56]. Zhang et al. [57] described in a pilot study how the combination of GnRH agonist and aromatase inhibitor have demonstrated promising long-term effects in young obese EC patients who wished to preserve their fertility. Fujimoto et al. found that a thin endometrium after repeated curettage had a negative effect on the endometrial receptivity to a pregnancy after conservative treatment for EC [58]. The risk of recurrence in patients submitted to conservative FST ranged from 14% in women with endometrial hyperplasia to 25% in women with carcinoma during the 39 months period studied [6]. In another study, the recurrence rate of EC reported after conservative treatment was between 30–40% from 4 to 66 months [5]. Despite successful hormonal treatment of EC, many young women still face subfertility due to preexisting metabolic disorders, such as PCOS. These women have a reduced rate of conception and live birth, due to risk factors such as obesity, metabolic syndrome, or chronic anovulation that also contributed to the development of EC. This subgroup of women showed a lower pregnancy rate due to their endometrial pathology. Insulin resistance and a body mass index > 25 kg/m^2^ have been associated with a higher complete response failure rate, longer duration of therapy, and increased rate of recurrence [59]. This group of women with overweight issues or obesity and insulin resistance also have a higher risk of adverse obstetric outcome or obstetric complications, and a higher infertility rate. It is important to change their lifestyle for both better oncological and fertility outcomes, since optimizing their health status with lifestyle interventions should improve their chance of becoming pregnant.

With the advance of assisted reproductive technology (ART), pregnancies in these sub-fertile women have been reported [60]. Chao et al. analyzed 50 women with AH/EIN/EC G1 IA: Group 1 (14 women) who had a pregnancy after in vitro fertilization, intracytoplasmic sperm injection, gamete intrafallopian transfer, or zygote intrafallopian transfer, and Group 2 (36 women) consisted of cases who had spontaneous conception or had ovulation induction with intrauterine insemination.

A higher rate of obstetric complications such as hemolysis, elevated liver enzymes and low platelets (HELLP) syndrome, gestational diabetes mellitus, and pregnancy-induced hypertension was reported in group 1. The reason why infertile women submitted to ART obtain worse obstetric outcomes might be the association of PCOS or other metabolic complications. This could also explain the increased number of gestational diabetes mellitus, which may be reflected by glucose intolerance and other metabolic disorders. Nevertheless, placental abruption, placental abnormalities, fetal chromosome, or structural abnormalities were insignificant between women who underwent ART and women who conceived spontaneously in this meta-analysis [60]. Park et al. reported the experience of 141 women with stage IA G1 EC. A total of 70 (49.6%) of the 141 patients tried to conceive, with 44 (62.9%) receiving fertility drugs. They reported that the use of fertility drugs was not associated with a higher incidence of cancer recurrence after successful FST in this study population. The spontaneous abortion rate, ectopic pregnancy rate, and preterm delivery rates in that cohort were 24%, 2.8%, and 11.5%, respectively [5]. Wang et al. retrospectively collected 67 patients diagnosed with EC/AH divided into three groups according to the treatment duration: group A ≤ 6 months, group B from 6–9 months, and group C > 9 months. No significant difference in the relapse rates was found between the three groups. Fertility rates and the time interval to pregnancy were not significantly different among the three groups. ART technique was associated with a higher pregnancy rate. Being overweight and obesity were associated with lower pregnancy rates [61]. The same conclusion that patients undergoing ART were more likely to become pregnant after FST was reported by Gallos in 2012, and Zhou in 2015 [62,63]. Those women who received FST had a similar 5-year disease free survival rate compared to those who did not, and women who had at least one live birth had a better free-disease survival with low recurrence rate [5]. It is recommended that ART should be started as soon as possible to improve pregnancy success, reduce time prior to definitive surgery, and minimize prolonged unopposed estrogen stimulation, which could also result in relapse and disease progression [62].

## 5. Discussion

EC rarely occurs in premenopausal age, with a prevalence correlated to the increase in a woman’s age, and an incidence between 5.0 and 6.6 per 100,000 women and 62.3 and 100.3 per 100,000 women, respectively before and after 50 years of age [2]. It is associated with a good prognosis, with a 5-year relative overall survival of 81.1%, which increases to 94.9% for patients affected by uterine-confined disease [2].

Historically, EC was divided in two clinical-pathological types: type 1, endometrioid- estrogen-related neoplasm associated with all causes of hyperestrogenism not balanced by progesterone/progestin presence (obesity, PCOS, metabolic syndrome). Nulliparity and infertility are classical risk factors for type 1 EC [64].

Type 1 covered 85% of ECs [65]. Type 2 is non-endometrioid, not related to hormones, and includes serous, clear cell, undifferentiated carcinomas, and malignant mixed Mullerian tumors [66] The traditional dualistic histopathologic classification is soon going to be abandoned, and a more heterogeneous disease is going to be recognized based on clinical and pathological factors reported by the European Society for Medical Oncology (ESMO) the European Society for Radiotherapy & Oncology (ESTRO) and the European Society of Gynaecological Oncology (ESGO) in a consensus published in 2016 [67], and on genetic and molecular classification, as reported in ESGO/ESTRO/ESP consensus published in 2021 (Table 1, Table 2) [39].

FST of EC patients seems to be feasible and safe in selected patients, affected by Grade1 endometrioid EC or by premalignant disease such EIN [4]. Thanks to the recent classification models by Colombo et al. and by Concin et al. the selection criteria could be improved and consider more clinical and biological targets to personalize EC patients’ treatment and counselling.

Some authors described predictive markers of response to FST such as histologic type, personal history of infertility, previous pregnancy, diabetes mellitus, PCOS, obesity, menstrual cycle characteristics, hormonal therapy, and age at diagnosis [30].

Genetic and epigenetic features have been emerging among the prognostic markers of EC [8,68,69]. These could drive clinicians to better select patients for a tailored treatment, in terms of surgical procedures and adjuvant therapies [70,71].

Balancing the risk benefits ratio for EC patients submitted to FST, it is important to consider the possibly increased risks for pregnancy and obstetric outcomes. Many FST could result in subfertility conditions and require ART treatments. Last year the European Society of Human Reproduction and Embryology (ESHRE) published a guideline dedicated to patients undergoing FST, demonstrating the growing sensitivity of the scientific community towards the quality of life and the reproductive potential of cancer patients [72]. Early involvement of fertility specialists during treatment planning is required to optimize opportunities for counseling and outcomes. It is therefore extremely important to carry out a well-planned preconception consultation about the increased maternal-fetal risks in a future pregnancy after FST in order to optimize the state of maternal health [51,73].

The new molecular classification proposed by Concin et al. plays a particularly important role for young patients suffering from EC as, in addition to predicting their prognosis, it could identify patients who might benefit from hormone therapy [9]. Some authors have assessed that the application of the ProMisE molecular classification has proved to be reliable also in young patients [74,75].

In particular, the MMRd patients showed later menarche compared with the MMS patients, showing an association between endocrine characteristics of EC patients and EC molecular-biological type [76]. Gan et al. described the association between MMRd and a significantly lower mean percentage of androgen receptor expression [77]. Some authors performed a retrospective molecular analysis on EC women younger than 40 years old, demonstrating that patients with MMRd type show high grade and high stage EC with low estrogen and progesterone receptors expression, concluding that this subset of patients would not be appropriate candidates for conservative management [78]. This finding was reinforced by a recent multicenter study involving a larger cohort of patients, showing an association between MMRd status and higher recurrence of AH/EC after initial regression [79].

The above considerations lead to the conclusion that a preliminary molecular analysis on an endometrial biopsy is necessary. Pathologists should systematically perform a molecular analysis on hysteroscopic biopsy samples, with the immunohistochemistry for the detection of p53 and mismatch repair proteins (MMR), and with the sequencing for the presence of the POLE mutation. It seems to be feasible, and some authors have reported a high concordance between endometrial biopsy or curettages and hysterectomy specimens regarding the molecular analyses [80].

Fertility counselling is imperative, each patient must be informed that FST is not the standard treatment. Only patients who strongly desire to preserve fertility should be treated conservatively, knowing that after childbearing completion, every EC patient will be submitted to definitive surgery including total abdominal hysterectomy with bilateral salpingo-oophorectomy, pelvic washing, and/or lymphadenectomy.

Careful selection of patients is mandatory to allow both oncological safety and an acceptable pregnancy rate.

EC patients submitted for FS treatments should be addressed in a referral center with an adequate multidisciplinary team. Oncologic and reproductive medicine should be strictly linked in this subset of patients, to ensure the highest possibility of cure and to preserve the reproductive function as well. Fertility sparing programs for oncofertility are based on several consolidated risk factors such as age, stage, histotype, and grading of the tumor. It would be challenging to consider emerging prognostic factors to tailor FST to these young oncological patients. Moreover, cancer patients can develop obstetric complications during pregnancy, as reported by some authors [60,81,82,83], both for the conception by ART and for the impact of surgical or medical treatments to which they have been submitted. Clinicians must offer these patients the possibility of being followed up in a high-risk pregnancy setting, to predict and anticipate eventual obstetric complications.

A larger amount of data, longer follow up periods, and data collection of pregnancy outcomes after FST are needed to improve the selection and satisfaction of a patient.

## 6. Conclusions

Young women with EC represent a group of patients who should be referred early on to a highly specialized center, with an experienced multidisciplinary team and a well-developed oncofertility path. The current selection of patients to submit to FST can be based on increasingly precise tools, such as molecular features of EC. Further studies are needed to evaluate the reliability of the molecular classification to select patients for FST having information both on oncological safety and on obstetric outcome.

## Figures and Tables

**Table 1:** Endometrial cancer ESMO/ESGO/ESTRO classification system, by Colombo et al. 2016 [67].

Risk Category	Risk Factor Status
Low	FIGO Stage I Endometrioid histotype grade 1–2 <50% myometrial invasion LVSI negative
Intermediate	FIGO Stage I Endometrioid histotype grade 1–2 ≥50% myometrial invasion LVSI negative
High-intermediate	FIGO Stage I Endometrioid histotype grade 3 <50% myometrial invasion regardless of LVSI status or FIGO Stage I Endometrioid histotype grade 1–2 LVSI unequivocally positive regardless of depth of invasion
High	FIGO Stage I Endometrioid histotype grade 3 ≥50% myometrial invasion regardless of LVSI status or FIGO Stage II or FIGO Stage III Endometrioid histotype no residual disease or Non-endometrioid histotype (Serous, clear cell, undifferentiated carcinoma or carcinosarcoma)
Advanced	FIGO Stage III residual disease or Stage IVA
Metastatic	FIGO Stage IVB
	(1)

**Table 2:** Endometrial cancer ESMO/ESGO/ESP classification system, by Concin et al. 2021 [8].

Risk Category	Risk Factor Status
Low	FIGO Stage I–II POLEmut no residual disease or FIGO stage IA MMRd/NSMP Endometrioid histotype grade 1–2 LVSI negative or focal
Intermediate	FIGO Stage IB Endometrioid histotype grade 1–2 LVSI negative or focal or FIGO stage IA MMRd/NSMP Endometrioid histotype grade 3 LVSI negative or focal or FIGO stage IA p53abn and/or non-endometrioid histotype (Serous, clear cell, undifferentiated carcinoma or carcinosarcoma)
High-intermediate	FIGO Stage I MMRd/NSMP substantial LVSI regrdless of grade and depth of invasion or FIGO Stage IB MMRd/NSMP Endometrioid histotype grade 3 regardless of LVSI status or FIGO Stage II MMRd/NSMP Endometrioid histotype
High	FIGO Stage III–IVA MMRd/NSMP Endometrioid histotype no residual disease or FIGO Stage I-IVA p53abn no residual disease or FIGO Stage I–IVA MMRd/NSMP serous, undifferentiated carcinoma or carcinosarcoma with myometrial invasion no residual disease
Advanced metastatic	FIGO Stage III–IVA residual disease any molecular type or Stage IVB any molecular type

## Data Availability

Not applicable.
